# A review of animal models for post-operative pericardial adhesions

**DOI:** 10.3389/fsurg.2022.966410

**Published:** 2022-09-12

**Authors:** Morgan A. Hill, O. Agata Walkowiak, William T. Head, Jennie H. Kwon, Minoo N. Kavarana, Taufiek Konrad Rajab

**Affiliations:** Division of Cardiothoracic Surgery, Medical University of South Carolina, Charleston, SC, United States

**Keywords:** adhesions, cardiac surgery, animal model, post-operative adhesions, pericardial adhesions

## Abstract

Post-operative pericardial adhesions remain a serious complication after cardiac surgery that can lead to increased morbidity and mortality. Fibrous adhesions can destroy tissue planes leading to injury of surrounding vasculature, lengthening of operation time, and increased healthcare costs. While animal models are necessary for studying the formation and prevention of post-operative pericardial adhesions, a standardized animal model for inducing post-operative pericardial adhesions has not yet been established. In order to address this barrier to progress, an analysis of the literature on animal models for post-operative pericardial adhesions was performed. The animal model, method used to induce adhesions, and the time to allow development of adhesions were analyzed. Our analysis found that introduction of autologous blood into the pericardial cavity in addition to physical abrasion of the epicardium caused more severe adhesion formation in comparison to abrasion alone or abrasion with desiccation (vs. abrasion alone *p* = 0.0002; vs. abrasion and desiccation *p* = 0.0184). The most common time frame allowed for adhesion formation was 2 weeks, with the shortest time being 10 days and the longest being 12 months. Finally, we found that the difference in adhesion severity in all animal species was similar, suggesting the major determinants for the choice of model are animal size, animal cost, and the availability of research tools in the particular model. This survey of the literature provides a rational guide for researchers to select the appropriate adhesion induction modality, animal model, and time allowed for the development of adhesions.

## Introduction

Post-operative pericardial adhesions remain an unsolved problem in cardiac surgery. Clinically, the most important aspects of post-operative pericardial adhesion formation are significantly increased morbidity and mortality during re-operative cardiac surgery. Re-operative cardiac surgery is particularly common in congenital cardiac surgery. An analysis of 2,555 cardiac re-operations at the Mayo Clinic revealed that iatrogenic injuries occurred in 9% of cases (1). Whereas the hospital mortality rate was 6.5% among patients without injury, this increased to 18.5% among those with injury; and the mortality rate was 25% when injury occurred during sternal division (1). The mechanism by which post-operative pericardial adhesions cause this dramatically increased mortality is obliteration of tissue planes. This puts vital structures such as the aorta, coronary arteries, right ventricle and right atrium at risk for injury during sternal re-entry and dissection. Apart from affecting patient outcomes, post-operative pericardial adhesions also have important implications for healthcare economics by significantly increasing operative time and healthcare costs.

For these reasons there is an urgent clinical need for improved strategies for prevention and treatment of post-operative pericardial adhesions. This is the focus of an intensive research effort underpinned by a wide variety of animal models. However, while animal models to study post-operative pericardial adhesions are legion, an analysis of the available animal models has not yet been performed. This is a critical barrier to progress in the field because the animal models employed are highly heterogeneous and results cannot be easily compared. Here we address this barrier to progress by analyzing the literature on animal models for post-operative pericardial adhesions.

## Methods

### Literature search

**Figure 1:**
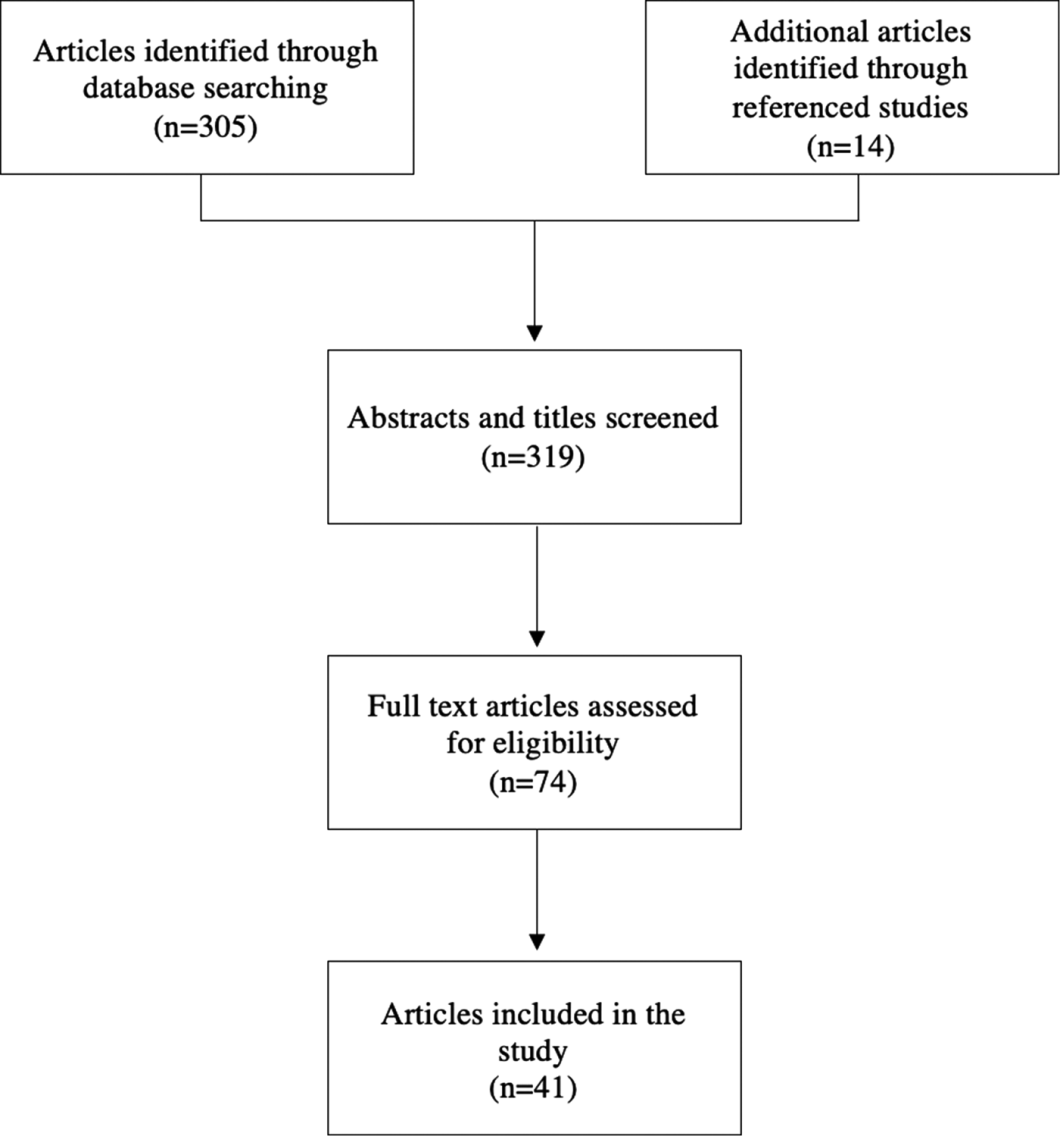
Methodology for the selection and exclusion of studies for analysis.

In order to determine a standardized animal model for studying pericardial adhesions, studies were evaluated to determine the most effective method to induce adhesion formation. A literature search for relevant articles was conducted using PubMed and Google Scholar from the inception of the respective databases (Figure 1). Search strategies were “pericardial adhesions”, “intrapericardial adhesions”, “adhesions” and “cardiac surgery”, “adhesions” and “heart surgery”, “pericardial barrier”, and “pericardial membrane”. Inclusion criteria for the search were animal studies published prior to 2021 that evaluated post-operative pericardial adhesions that included a negative control group in which no intervention was used to prevent adhesions. Exclusion criteria were studies that were published in a language other than English and studies that did not provide numerical data nor an adhesion score based on a finite scale.

### Data analysis

The appropriate studies were selected, and the negative control groups of each study were compared to determine which models produced the most severe post-operative pericardial adhesions. The variables analyzed included animal model, number of animals in the control group, method used to induce pericardial adhesions, length of time allowed for adhesion formation, and grading of the adhesions. In order to account for the various methods to grade adhesions used by each study, all reported adhesion scores were converted to a standardized severity score out of 10 which allowed for comparison of adhesion severity between all studies (Equation 1). A mean severity score and standard deviation were calculated for each study. A single pooled mean and standard deviation was calculated for studies that used a similar animal model or method (Equations 2, 3) (2). Statistical analyses and graphing were performed in Prism v5 and Python with the Matplotlib and NumPy libraries.

**Equations 1–3:** Formulas used to calculate standardized adhesion score, pooled mean, and standard deviation.

$$Standardized\;severity\;score=\frac{given\;adhesion\;score}{maximum\;adhesion\;score}\;\;\times \;\;10$$
$$x_{c}=\frac{m\cdot x_{a}+n\cdot x_{b}}{m+n}$$
$$s_{pooled}=\sqrt{\frac{\left(n_{1}-1\right)s_{1}^{2}+\left(n_{2}-1\right)s_{2}^{2}+\ldots +\left(n_{k}-1\right)s_{k}^{2}}{n_{1}+n_{2}+\ldots +n_{k}-k}}$$

## Results

A total of 41 studies involving animal models for post-operative pericardial adhesions were identified. One study included both a pig and rabbit model which were evaluated separately (3).

### Animal species

The animal species used were mouse (2% of studies, *n* = 1 study with *n* = 4 total animals) (4), rat (7%, *n* = 3 studies with *n* = 28 total animals) (5–7), hamster (2%, *n* = 1 study with *n* = 11 total animals) (8), rabbit (44%, *n* = 18 studies with *n* = 235 total animals) (3, 9–25), canine (19%, *n* = 8 studies with *n* = 66 total animals) (26–33), pig (19%, *n* = 8 studies with *n* = 53 total animals) (3, 34–40), and sheep (7%, *n* = 3 studies with *n* = 15 total animals) (41–43) (Table 1).

**Table 1:** The number of studies and total number of animals evaluated for each animal model.

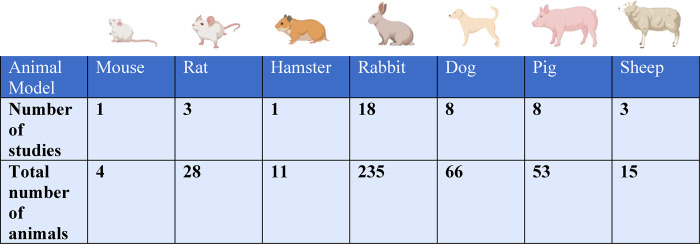

**Figure 2:**
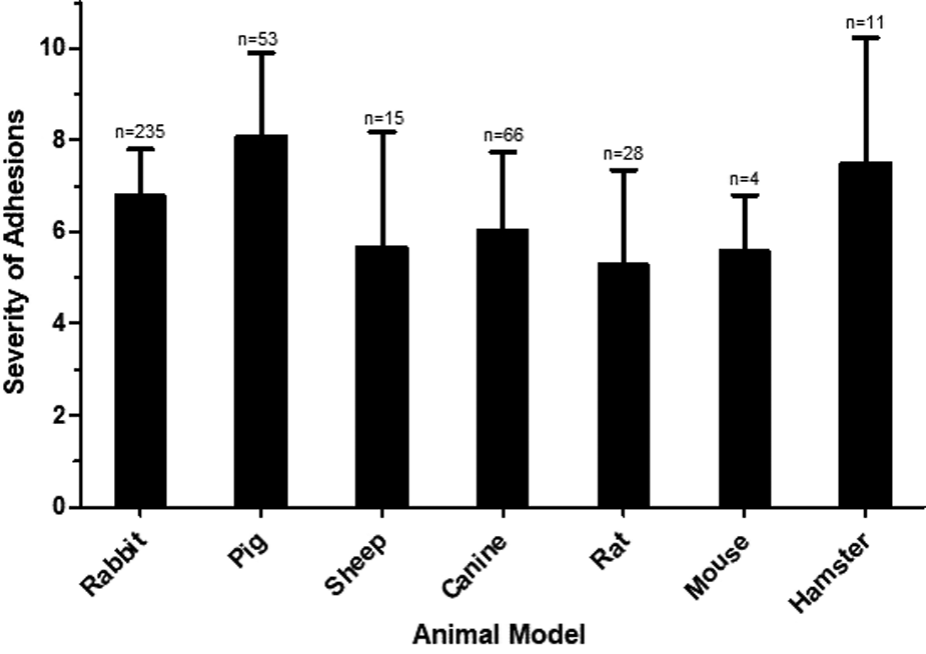
Severity of adhesions in various animal models. The mean severity score for each animal model is shown, with error bars representing standard deviation. N represents the number of animals used in each model.

The tendency of severe postoperative adhesions to form in various animal models irrespective of the method of traumatization was compared (Figure 2). The pig models demonstrated more severe adhesion formation in comparison to all other animal models with the exception of hamster (vs. rabbit *p* < 0.0001; vs. canine *p* < 0.0001; vs. sheep *p* < 0.0001; vs. rat *p* < 0.0001; vs. mouse *p* < 0.0196). The rabbit model was most frequently used and showed greater adhesion severity compared to canine and rat (vs. canine *p* = 0.0045; vs. rat *p* < 0.0001).

### Modalities for adhesion induction

**Figure 3:**
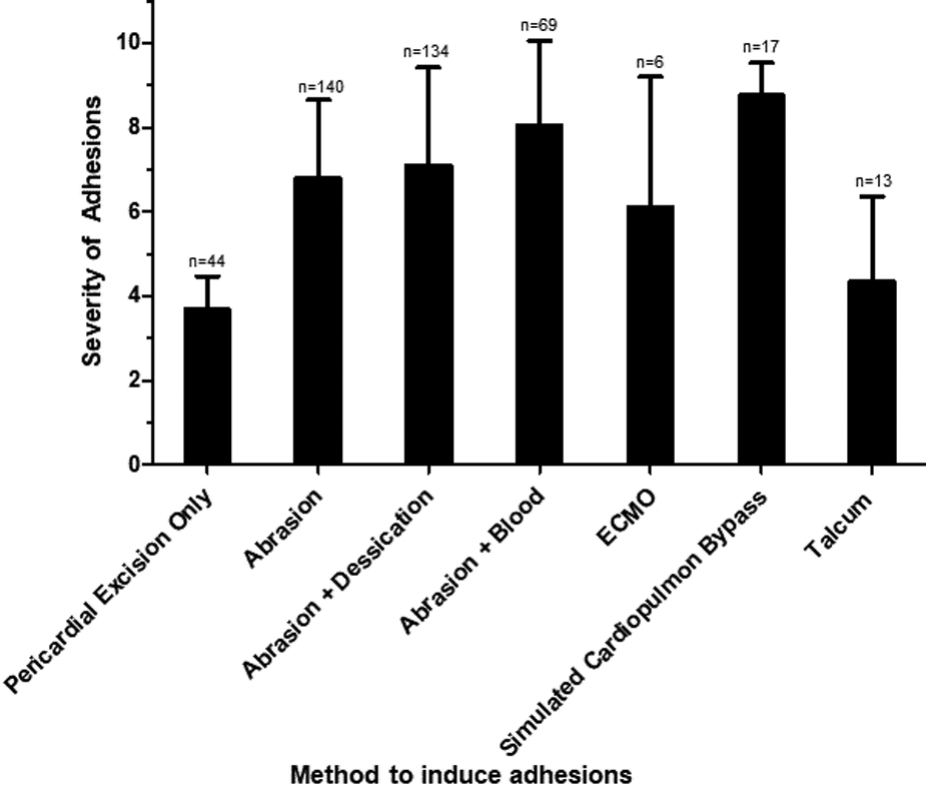
Effect of method of adhesion induction on severity of adhesions. The mean severity score for each method is shown, with error bars representing standard deviation. N represents the number of animals used per method.

The modalities used to induce post-operative pericardial adhesions in animal models were: pericardial excision only, physical abrasion of the epicardium, abrasion of the epicardium in addition to desiccation, abrasion of the epicardium with introduction of autologous blood into the pericardial cavity, injection of talcum, institution of extracorporeal membrane oxygenation (ECMO) and simulated cardiopulmonary bypass cannulation through various dissection and suturing techniques. Each method's effectiveness at inducing post-operative adhesion formation was analyzed by comparing the severity of pericardial adhesions formed (Figure 3). Animals that only underwent pericardial excision had significantly less severe post-operative adhesions than all of the other methods with the exception of institution of ECMO and injection of talcum (vs. abrasion *p* < 0.0001; vs. abrasion and desiccation *p* < 0.0001; vs. abrasion and blood *p* < 0.0001; vs. simulated bypass *p* < 0.0001). Introduction of autologous blood into the pericardial cavity in addition to physical abrasion caused the formation of more severe adhesions in comparison to abrasion alone or abrasion with desiccation (vs. abrasion alone *p* = 0.0002; vs. abrasion and desiccation *p* = 0.0184). Injection of talcum was less effective at inducing severe adhesions compared to all of the abrasion models and simulation of cardiopulmonary bypass and was not significantly better at inducing severe adhesions than performing pericardial excision alone (vs. abrasion *p* = 0.0004; vs. abrasion and desiccation *p* < 0.0001, vs. abrasion and blood *p* < 0.0001, vs. simulated bypass *p* < 0.0001, vs. pericardial excision only *p* = 0.946).

## Comment

Animal models to study post-operative pericardial adhesions are numerous, but highly heterogeneous. Our systematic analysis of the literature identified a total of 42 animal models. We analyzed these models with regards to the mechanism for adhesion induction, the animal species used, and the timing of analysis.

### Mechanisms for adhesion induction

Pericardial adhesions are a physiologic response to mediastinal trauma that aims to seal off vascular injuries and thus ensure survival of the organism. This involves a cascade of biochemical and cellular events with an imbalance of inflammatory responses, coagulation mechanisms, angiogenesis and fibrinolysis (44). Broadly, pericardial adhesion formation is initiated by the deposition of fibrin, and the formation of fibrin bridges between mediastinal tissues. Subsequent cellular organization of these fibrin bridges by fibroblast migration and vascularization under the influence of inflammatory growth factors results in the formation of permanent adhesions (45–47). This cascade can be triggered by a wide variety of traumatic stimuli.

In the literature, seven different methods were used to induce postoperative pericardial adhesions, namely, pericardial excision only, physical abrasion of the epicardium, abrasion of the epicardium in addition to desiccation, abrasion of the epicardium with introduction of autologous blood into the pericardial cavity, and injection of talcum. In an effort to more closely replicate the conditions of cardiothoracic surgery, one study instituted ECMO (42) and others simulated cardiopulmonary bypass cannulation through various dissection and suturing techniques (31, 37, 39). Mitchel et al. hypothesized that while mild mesothelial injury alone and blood clots inside an uninjured serosal cavity are not sufficient to induce pericardial adhesions on their own, the combination of these conditions can facilitate adhesion formation (31). Our findings support this conclusion, as introduction of autologous blood into the pericardial cavity in addition to physical abrasion was found to induce more severe post-operative adhesions compared to abrasion alone or abrasion with desiccation (vs. abrasion alone *p* = 0.0002; vs. abrasion and desiccation *p* = 0.0184).

In contrast, animals that only underwent pericardial excision had significantly less severe post-operative adhesions than all of the other methods with the exception of institution of ECMO and injection of talcum, indicating that pericardial excision alone is not sufficient to induce post-operative adhesions that are representative of clinical conditions.

While administration of talcum into the pericardial space allows for a simpler way to induce adhesions without performing a thoracotomy, we found injection of talcum was less effective at inducing severe adhesions compared to all of the abrasion models and simulation of cardiopulmonary bypass, and was not significantly better at inducing severe adhesions than performing pericardial excision alone (vs. abrasion *p* = 0.0004; vs. abrasion and desiccation *p* < 0.0001, vs. abrasion and blood *p* < 0.0001, vs. simulated bypass *p* < 0.0001, vs. pericardial excision only *p* = 0.946). However, this finding may in part be due to the small sample size of this adhesion model (*n* = 2 studies, *n* = 13 animals).

An important consideration in the development of pericardial adhesions is the method of entry into the mediastinum, such as *via* thoracotomy, sternotomy, or other less invasive techniques. However, this was not the focus of our study and thus is a limitation of the presented work.

### Animal species to study post-operative pericardial adhesions

Animal species for surgical models are typically categorized as small animals (mouse, rat, hamster and rabbit) and large animals (dog, sheep and pig). Advantages of small animals are the ability to have a higher number of replicates, whereas the heart of large animals is more similar in size to human hearts. We found that the differences in adhesion severity in all animal species was similar (Figure 2). Therefore, the major determinant for the choice of the model are animal size, animal cost, and the availability of research tools in the particular model.

By far the most commonly used species to study post-operative pericardial adhesions were rabbits (Table 1). This species was used in almost half of studies (*n* = 18/41 studies), which included over half of all animals (*n* = 235/412 animals). The most likely reason for this is that rabbits are the largest of the small animal models. Therefore, this model combines the advantages of the ability to perform more replicates with the advantages of larger operative structures. The next most commonly used species were the large animals dog (*n* = 8 studies) and pig (*n* = 8 studies). This indicates that the major focus of many of the studies was to test surgical approaches or mechanical barrier methods to prevent adhesions. Both are relatively expensive animal models. In contrast, only a relatively small number of studies used the small animals mouse, rat or hamster. This is surprising because the research tools to study genetic and molecular mechanisms are by far the most advanced in these animal models. We anticipate that as research moves from mechanical barriers towards molecular therapeutics, the mouse and rat models will become progressively more important.

### Timing of analysis

The kinetics of post-operative adhesion formation is well studied in the peritoneum. The peritoneum requires approximately 7 days to regenerate the mesothelial lining irrespective of the size of the peritoneal injury, as the entire surface of the peritoneum mesothelializes from deep to superficial. This differs from injuries to the skin, which epithelialize from the borders of the defect inward (48). In contrast, healing of pericardial and epicardial injuries are poorly studied. Therefore, the time window during which the pericardium and epicardium are vulnerable to adhesion formation is unclear. Our review found that the earliest time for analysis of pericardial adhesions was 10 days. The most common time course for analysis was 2 weeks. Most studies examined adhesions within the first 8 weeks. The latest time-point for analysis was 12 months. More recently, a study analyzed the formation of pericardial adhesion in relation to angiogenesis in mice models in a 7 day long study (49). This suggests that molecular components of pericardial adhesions may begin before 10 days. Future studies should monitor for pericardial adhesions as early as a few days after induction. Additionally, in the future, investigators may consider analyzing adhesion formation at 2 weeks or earlier since later analysis increases the costs of the experiment.

### Mechanisms for adhesion prevention

Pericardial adhesions continue to be a major complication of cardiac surgery. Currently there are multiple methods to prevent adhesions. Mechanical barriers include solid polymers, gels, and liquids (44). Ideally, a mechanical barrier would be present throughout the entire healing process as well as resistant to the immune processes of adhesion formation. It should also be removable or degradable once healing is complete. Antiadhesive agents have also been used; these can work to prevent the adhesion formation pathways or enhance the fibrinolytic ones (44). Finally, physical therapy and movement has been linked to decreased adhesion in joints and the abdomen (44). We have previously described a systematic review of the efficacy of currently available adhesion barriers (50).

## Conclusions

While there are many researchers utilizing animal models to investigate post-operative pericardial adhesion formation and prevention, a standardized pericardial adhesion model does not yet exist. Therefore, direct comparisons of interventions to treat post-operative pericardial adhesions are difficult. Our review provides the first survey of the described pericardial adhesion models. This will help investigators make rational choices when choosing a model for pericardial adhesions. Ideally, studies of pericardial adhesions should employ standardized models to allow easy interpretation and comparison. Furthermore, the standardization of pericardial adhesion induction would allow for the study of adhesion prevention techniques.

## Classifications

Animal model, surgery complications, congenital heart surgeon, cardiac pathologic anatomy.
